# Preventive treatment response associated with migraine aura subtypes in a Thai population

**DOI:** 10.3389/fnhum.2022.1065859

**Published:** 2023-01-09

**Authors:** Thanin Asawavichienjinda, Robin James Storer

**Affiliations:** ^1^Chulalongkorn Comprehensive Headache Center, King Chulalongkorn Memorial Hospital, Department of Medicine, Faculty of Medicine, Chulalongkorn University, Bangkok, Thailand; ^2^Office of Research Affairs, Faculty of Medicine, Chulalongkorn University, Bangkok, Thailand

**Keywords:** migraine disorders (MeSH term), migraine with aura, preventive medication, anticonvulsants, quality of life

## Abstract

**Introduction:**

Some studies indicate a different response to treatment between migraine patients with and without aura.

**Objectives:**

To determine whether aura, or simple or complex aura subtypes, are clinical markers predicting response to preventive treatment.

**Methods:**

Conducted a retrospective cohort study at a headache clinic in a tertiary referral hospital. We included data from patients registered from 1 November 2014, to 30 June 2022, having migraine with or without aura, or with simple or complex aura, and who had received migraine preventive treatments with at least 3 months follow-up. The primary outcome was a response to preventive treatment defined as at least a 50% reduction from a baseline of monthly migraine or headache days (MMDs/MHDs). Secondary outcomes were improvement in quality of life and disability scores.

**Results:**

For migraine patients with (45) and without (123) aura who took a migraine preventive with at least 3 months follow-up; except for median age, which was older for patients without aura, baseline sex, comorbidity, and migraine data were without significant difference including median history of migraine, chronic migraine subtype, chronic migraine with medication-overuse headache, median or mean MMDs/MHDs, number of preventive medications used, or migraine preventive medication inhibiting spreading depolarizations. Treatment outcomes at 3 and 6 months follow-up were not significantly different between migraine patients with and without aura, or with simple and complex aura, but tended to be greater in those with aura and those with complex aura. After adjustment for baseline comorbidity, migraine subtypes, aura subtypes, the number of preventives used, history of migraine, and MMDs/MHDs, we found no significant differences in 30% and 50% reduction from baseline of MMDs/MHDs in 3 or 6 months or most recent follow-up.

**Conclusions:**

Preventive treatment response tended to be associated with migraine aura subtypes. We found preventive treatment response tended to have more favorable outcomes in those with aura, especially those with complex aura.

## 1. Introduction

Migraine treatment consists of two approaches, treatment of acute attacks, and preventive treatment, which aims to reduce the frequency, severity, and duration of migraine attacks. The disorder can be categorized into two major subtypes: migraine with aura and migraine without aura. Because of the possibly different pathophysiology and etiology of migraine between the subtypes (Hansen and Charles, 2019), treatment responses may be different. From preclinical studies, preventive medications that control cortical spreading depression, including topiramate and valproate (Kaube and Goadsby, 1994), may be more likely to control headache frequency in migraine with aura (Ayata et al., 2006; Bogdanov et al., 2011). A systematic review of pharmacological agents against spreading depolarizations found valproate and topiramate significantly inhibited spreading depolarization (Klass et al., 2018). In clinical practice, one study found that migraine aura predicts treatment response to rimabotulinum toxin A (Grogan et al., 2013), but another study with onabotulinum toxin A did not support that finding (Jakubowski et al., 2006). Because migraine aura has heterogeneous manifestations, a scale for assessment of the complexity of migraine aura called the Migraine Aura Complexity Score has been proposed and developed (Petrusic et al., 2019a). The scale was applied to patients with migraine aura who can be categorized into three groups, including those with migraine with simple aura, migraine with moderately complex aura, and migraine with complex aura, which may reflect differences in the thickness of the cerebral cortex (Petrusic et al., 2019b). The extent of the thickness of the cerebral cortex may lead to different responses to preventive treatment. Data for treatment responses in patients with migraine with and without aura, or with simple or complex aura, appears scant. Therefore, we conducted a retrospective cohort study using data from a specialist center at a large tertiary referral hospital. We sought to examine the treatment responses in migraine patients with and without aura, and the treatment responses with topiramate or valproate or anti-calcitonin gene-related peptide (CGRP) monoclonal antibodies, medications inhibiting spreading depolarizations, and with any preventive medications in migraine patients with simple and with complex aura to determine any difference or correlation between them. Simple aura includes simple visual or somatosensory symptoms alone. Complex aura includes visual and other symptoms, or somatosensory and dysphasic symptoms, or complex visual or somatosensory aura alone.

## 2. Materials and methods

### 2.1. Design

This retrospective cohort study was conducted at the Chulalongkorn Comprehensive Headache Center, King Chulalongkorn Memorial Hospital, a public general and tertiary referral hospital in Bangkok, Thailand, serving as a teaching hospital for the Faculty of Medicine, Chulalongkorn University. With an in-patient capacity of 1,435 beds, it is one of the largest hospitals in Thailand. The headache center has registered patients and created a case-record form for gathering patients’ demographic and socioeconomic data and headache information on every visit since 3 January 2007. The present study included data from all patients registered between 1 November 2014, and 30 June 2022, and was approved by the Institutional Review Board of the Faculty of Medicine, Chulalongkorn University (IRB No. 0644/65).

### 2.2. Population

We included patients with migraine according to the International Classification of Headache Disorders (ICHD), 2nd edition (Headache Classification Subcommittee of the International Headache Society [IHS], 2004), ICHD, 3rd edition (beta version), and 3rd edition (Headache Classification Committee of the International Headache Society [IHS], 2013, 2018), in the present study. The diagnostic criteria for migraine without aura are the same for the three editions of the ICHD. However, the diagnostic criteria for migraine with aura are not the same. Migraine with aura according to the 2nd edition, is classified into six subforms including typical aura with visual, sensory, and dysphasic speech disturbance as either positive or negative features with migraine headache; typical aura with non-migraine headache; typical aura without headache; familial hemiplegic migraine; sporadic hemiplegic migraine; and basilar-type migraine, but not including retinal migraine. The diagnostic criteria for migraine with aura from the 3rd edition, beta version combines visual, sensory, speech or language disturbance, motor, brainstem, and retinal disturbance, and adds a criterion of unilateral aura symptom. The diagnostic criteria for migraine with aura from the 3rd edition adds a criterion of the positive aura symptom to the beta version.

The inclusion criteria were as follows: age between 15 and 80 years; taking preventive treatment; at least 3 months follow-up. The exclusion criteria were as follows: other primary and secondary headaches, but not medication-overuse headaches. Patients in this study were categorized into two groups, with aura or without aura, and with simple or complex aura. Potential chronic migraine and probable medication-overuse headache from the 2nd edition of the ICHD, and chronic migraine with medication-overuse headache from the beta version and the 3rd edition of the ICHD were combined to form a group with chronic migraine and medication-overuse headache.

### 2.3. Outcomes measurement and data collection

The primary outcome was a reduction from the baseline of monthly migraine/headache days (MMDs/MHDs) at month 3 for migraine patients with and without aura. The secondary outcome was a reduction from the baseline of MMDs/MHDs at month 3 for migraine patients with simple and complex aura. Other secondary outcomes were as follows: at least 30 and 50% reduction from baseline of MMDs/MHDs at month 3; a reduction from baseline of MMDs/MHDs at month 6, and at most recent follow-up; at least 30 and 50% reduction from baseline of MMDs/MHDs at month 6, and at most recent follow-up; quality of life score measured by using the Thai version of the Migraine-Specific Quality of Life Questionnaire (MSQ) version 2.1 (Asawavichienjinda et al., 2017) changed from baseline at months 3, 6, and at most recent follow-up; migraine disability score measured by using the Thai version of the Migraine Disability Assessment (MIDAS) questionnaire (Asawavichienjinda et al., 2020) changed from baseline at months 3, 6, and at most recent follow-up for migraine patients with and without aura and with simple and complex aura. Additional secondary outcomes were subgroup analysis of patients with simple and complex aura and preventive treatment response with antiepileptic drugs, including topiramate or valproate and anti-CGRP monoclonal antibodies, and a correlation between the complexity of aura and the success of preventive treatment. Data were gathered from case records. The data from eligible patients were included from consecutive registrations from June 30, 2022, and progressively less recently until they reached the expected number for migraine with and without aura. MHDs were applied to the chronic migraine and chronic migraine with medication-overuse headache groups because the headaches were not categorized as migrainous or not. The characteristics of migraine aura were collected by semistructured interviews with the following questions. “Do you have any warning symptoms 5 to 60 minutes before migraine headaches or during headaches? If ‘yes,’ please describe,” and the symptoms were recorded in the case record; if “no,” the following questions were asked. “Do you have any abnormal visual symptoms, or any abnormal sensation at fingers, hands, arms, face, or around the mouth or abnormal speech, slurred speech, difficulty in speaking or trouble finding word? If ‘yes,’ please describe.” Migraine with aura is categorized into two subtypes, including simple or complex aura, according to the description by Petrusic et al. (2019a). A simple visual aura is considered the following: flashes of bright light, sparkles of light, stars, zig-zag lines, snow falling or scotoma, and a complex visual aura includes a perceptual distortion of the size or shape of an object or change in color perception or fractured vision. A simple somatosensory aura includes tingling, paresthesia, numbness, or both, and a complex somatosensory aura includes peculiar sensations unilaterally in the hands, arms, face, and tongue. Language/speech aura (dysphasic disturbance), including difficulty in speaking or communication, slurred speech, trouble finding a word, impaired memory, apraxia, or ideational agnosia, are classified as complex aura, as well as simple visual aura followed by a somatosensory aura or visual or a somatosensory aura followed by a language/speech aura. The present study did not use a structured interview following the Migraine Aura Complexity Score (Petrusic et al., 2019a,b).

### 2.4. Statistical analysis

Descriptive statistics were applied for data summary as follows: proportion or percentage for categorical data; mean and standard deviation for continuous data with normal distribution; or median and interquartile range for continuous data with non-normal distribution. Inferential statistics were applied as follows: we used a chi-square or Fisher exact test to compare dichotomous data between the two groups, an unpaired *t* test to determine mean differences, and a Mann–Whitney *U* test to determine median differences. Multivariate analyses using log-binomial logistic regression were applied to adjust for treatment effect modifiers, including the number of preventive medications used at baseline, grouped into two categories: monotherapy and polytherapy; migraine subtypes, grouped into two categories: episodic and chronic migraine, or chronic migraine with medication-overuse headache; aura subtypes, grouped into two categories: simple or complex aura; and comorbidity, grouped into two categories, that is, with or without in a binary manner, to evaluate the treatment outcomes, including a 30% and 50% reduction of MMDs/MHDs at months 3, 6, and at most recent follow-up. The correlation between aura subtypes and the success of preventive treatment was applied using a contingency coefficient. A Bonferroni *post hoc* procedure was applied for a family-wise error rate due to multiple analyses. The sample size for migraine patients with aura and without aura was calculated with a minimum treatment response of 20% in the migraine patients without aura, an anticipated relative risk of 2.5, *P* < 0.05, and a power of 80%. The ratio of migraine patients with aura; to patients without aura, was 1:3. The sample size for migraine patients with aura was 39, and for migraine patients without aura was 117. We excluded cases where data was missing.

## 3. Results

**FIGURE 1:**
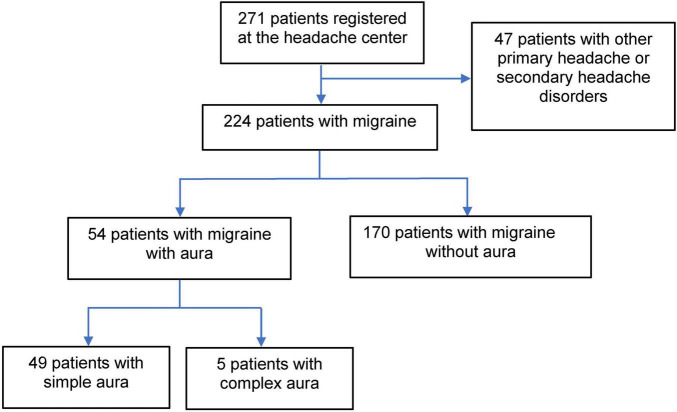
Flow chart for inclusion of patient data.

Between 1 November 2014, and 30 June 2022, 271 patients registered at our headache center. Of these 271 patients, 47 were diagnosed with secondary headaches or other primary headache disorders. Of the 224 patients with migraine, 54 (24.1%) had migraine with aura, and 170 (75.9%) had migraine without aura. Of the 54 migraine patients with aura, 49 had a simple aura, and 5 had a complex aura. The simple aura was as follows: simple visual aura for 41 (83.7%) and simple somatosensory aura accounted for 8 (16.3%) patients (Figure 1). The simple visual aura (41) included a blurred spot 26, sparkles of light or stars 6, flashes of bright light 3, zig-zag lines 3, flashes and then scotoma 2, and rainbow effects 1. The simple somatosensory aura (8 patients) included numbness in the hand for 4 patients and tingling in the hand for 4 patients. The complex aura (5 patients) was as follows: sparkles of light or stars and then numbness in the hand in 1 patient, and then numbness in hand and tongue in 1, numbness in hand and then slow speaking in 1, tingling in the hand and then difficulty in speaking 1, and motor weakness in 1. Of the 224 patients with migraine, 119 (53.1%) had comorbidity, including hypertension (33), allergic rhinitis (30), dyslipidemia (24), peptic ulcer (11), depression (9), asthma (9), gastroesophageal reflux disease (8), diabetes (7), thyroid disease (6), dizziness (4), ischemic heart disease (3), hypotension (2), and other comorbidities as follows: thyroid cancer (2), obstructive sleep apnea (2), irritable bowel syndrome (1), and stroke (1). Some migraine patients had more than one comorbidity. Of the 54 patients with aura, only 6 (11.1%) did not take a migraine preventive medication. Of the 6 migraine patients with aura, but not taking a migraine preventive medication, 5 (9.3% of migraine patients with aura) had 2–4 MMDs/MHDs, and one had 12 MMDs/MHDs but refused to take any medicines. Of the 170 migraine patients without aura, 28 (16.5%) did not take a migraine preventive medication. Of the 28 migraine patients without aura who were not taking a migraine preventive medication, 17 (10.0% of the migraine patients without aura) had 1–4 MMDs/MHDs, 6 (3.5% of the migraine patients without aura) had 7–12 MMDs/MHDs, and 5 (2.9% of the migraine patients without aura) had 16–28 MHDs/MHDs. We found no significant difference (*P* = 0.79) between migraine patients with aura (9.3%) and without aura (10.0%) in the proportion of patients without indication for a migraine preventive medication, equal to 4 MMDs/MHDs or less.

For migraine patients with and without aura who took a migraine preventive medication, except for the median age, which was older for migraine patients without aura, baseline sex, comorbidity, and migraine data were without significant differences, including the median history of migraine, chronic migraine subtype, chronic migraine with medication-overuse headache, median or mean MMDs/MHDs, median Thai-version MSQ version 2.1 score, median Thai-version MIDAS score, the number of preventive medications used, or migraine prevention inhibiting spreading depolarizations used (Table 1). The comorbidity in migraine patients with aura (27, 56.3%) was as follows: allergic rhinitis (8), asthma (5), hypertension (4), dyslipidemia (4), thyroid cancer (3), ischemic heart disease (3), and other comorbidities including stroke (1), depression (1), and dizziness (1). The comorbidity in migraine patients without aura (81, 57%) was as follows: hypertension (28), allergic rhinitis (19), dyslipidemia (19), peptic ulcer (11), depression (7), diabetes (7), gastroesophageal reflux disease (6), thyroid disease (4), asthma (3), and other comorbidities including hypotension (2), dizziness (2), irritable blow syndrome (2), obstructive sleep apnea (2), and thyroid cancer (1). Some migraine patients had more than one comorbidity. In detail, aura characteristics included visual phenomena for 36 patients (75.0%), somatosensory aura for 8 (16.7%), visual and then somatosensory aura for 1 (2.1%), somatosensory and then dysphasia for 2 (4.2%), and motor weakness aura for 1 (2.1%). Medications for migraine prevention at baseline for monotherapy included antiepileptic drugs (91 patients, 47.9%), antidepressants (35 patients, 18.4%), anti-CGRP monoclonal antibodies (11 patients, 5.8%), beta-blockers (4 patients, 2.1%), calcium channel blockers (4 patients, 2.1%), muscle relaxants (1 patient, 0.5%), and nutraceutical (7 patients, 3.7%). The antiepileptic drugs included topiramate, valproic acid, gabapentin, pregabalin, and zonisamide. The antidepressants included amitriptyline, nortriptyline, venlafaxine, and fluoxetine. Beta-blockers included propranolol, metoprolol, and atenolol. Calcium channel blockers included flunarizine. Muscle relaxants included tizanidine and eperisone. Nutraceutical included vitamin B2.

**TABLE 1:** Baseline age, sex, and migraine data for migraine patients with simple or complex aura and without aura taking migraine preventive medication.

Data	Migraine with aura (*n* = 48)			Migraine without aura (*n* = 142)	*P* (with vs. without aura)	*P* (simple vs. complex aura)
	Overall (*n* = 48)	Simple (*n* = 44)	Complex (*n* = 4)			
Median age, years (IQR)	36.5 (30–49)	33.5 (30-49)	42.0 (38.3-48)	46.0 (36–54.3)	0.012*	0.42
Female sex, *n* (%)	40 (83.3)	36 (81.8)	4 (100)	119 (83.8)	0.94	NA
Comorbidity presence, *n* (%)	27 (56.3)	24 (54.5)	3 (75.0)	81 (57.0)	0.94	0.79
Median history of migraine headache, years (IQR)	5.0 (2.1–10)	5.0 (2.1-10)	10 (4-17.5)	6.3 (2–10.5)	0.41	0.34
Chronic migraine subtype, *n* (%)	17 (35.4)	15 (34.1)	2 (50.0)	54 (38.0)	0.75	0.93
Chronic migraine with MOH	5 (10.4)	4 (9.1)	1 (25.0)	14 (9.9)	0.91	0.89
Median MMDs/MHDs (IQR)	16.6 (8–28)	12 (8-28)	28 (16-28)	14.0 (8–28)	0.99	0.11
Mean MMDs/MHDs	16.6 (9.1)	15.9 (9.0)	24 (8.0)	16.7 (9.0)	0.95	0.09
Median Thai-version MSQ v. 2.1 score (IQR)	60.1 (47.2–72.9)	63.6 (48.3–77.9)	51.5 (45–57.9)	62.9 (48.6–80.4)	0.80	0.25
Median Thai-version MIDAS score (IQR)	4 (0–9.3)	4 (0–9.3)	3 (0–11.3)	5.4 (0–5)	0.16	0.78
Number of preventive medications, *n* (%) ∙ Monotherapy ∙ Polytherapy	40 (83.3) 8 (16.7)	37 (84.1) 7 (15.9)	3 (75.0) 1 (25.0)	114 (80.3) 28 (19.7)	0.77	0.97
Migraine prevention inhibiting spreading depolarizations used, *n* (%)	19 (39.6)	19 (43.2)	0 (0)	51 (35.9)	0.78	NA

MIDAS, migraine disability assessment questionnaire; MMDs/MHDs, monthly migraine/headache days; MOH, medication-overuse headache; MSQ, migraine-specific quality of life questionnaire.

For migraine patients with simple and complex aura who took a migraine preventive medication, baseline age, sex, comorbidity, and migraine data were without significant differences, including the median history of migraine, chronic migraine subtype, chronic migraine with medication-overuse headache, median or mean MMDs/MHDs, median Thai-version MSQ version 2.1 score, median Thai-version MIDAS score, the number of preventive medications used, or migraine prevention inhibiting spreading depolarizations used (Table 1). However, the proportion of comorbidity presence and chronic migraine with medication-overuse headache was higher in the complex aura group compared to simple aura group, as well as median history of migraine headache, median or mean monthly headache days, which was longer or greater in the complex aura group. Migraine patients with simple aura taking migraine prevention inhibiting spreading depolarizations (43.2%) were more prevalent than those with complex aura (0).

During the treatment follow-up, 45 of the 48 migraine patients with aura, and 123 of the 142 migraine patients without aura had at least 3 months follow-up. Baseline age, sex, migraine data, and treatment outcomes for 3-month follow-up including a mean reduction from baseline of MMDs/MHDs, 30 and 50% reduction from baseline of MMDs/MHDs, mean improvement from baseline of Thai-version MSQ version 2.1, and mean reduction from baseline of Thai-version MIDAS score at 3, 6 months, and at most recent follow-up, were not significantly different between migraine patients with and without aura, but tended to be greater in those with migraine with aura (Table 2). For the outcomes of migraine patients with complex aura, mean reduction from baseline of MMDs/MHDs at 3, 6 months, and at most recent follow-up, and 30% and 50% reduction from baseline of MMDs/MHDs at 3 months follow-up had greater improvement with a relative risk of 1.46 (95% CI 1.19–1.80) and of 1.28 (95% CI 0.69–2.39), respectively, compared with those migraine patients with simple aura but no significant difference (Table 2). The correlation between the complexity of migraine and preventive treatment response had a very-weak-to-weak relationship (Table 3). A subgroup analysis of the complexity of aura and preventive treatment response with medications inhibiting spreading depolarizations was not analyzed because no migraine patients with complex aura took such medications. After adjustment for baseline migraine subtypes, aura subtypes, the number of preventive medications used, and comorbidity, we found no significant differences in 30 and 50% reduction from baseline of MMDs/MHDs in 3 or 6 months or most recent follow-up (Table 4).

**TABLE 2:** Baseline age, sex, migraine data, and treatment outcomes for 3 months follow-up for migraine patients with simple or complex aura and without aura.

Data	Migraine with aura (*n* = 45)			Migraine without aura (*n* = 123)	*P* (with vs. without aura)	*P* (simple vs. complex aura)
	Overall (*n* = 45)	Simple (*n* = 41)	Complex (*n* = 4)			
**Baseline data**						
Median age, years (IQR)	38.0 (30–49)	34 (29.5–49)	42 (38.3–48)	45.0 (36–55)	0.024*	0.43
Female sex, *n* (%)	39 (86.7)	35 (85.4)	4 (100)	102 (82.9)	0.73	NA
Comorbidity presence, *n* (%)	24 (53.3)	21 (51.2)	3 (75)	68 (55.3)	0.96	0.61
Median history of migraine headache, years (IQR)	5.0 (2–10)	5 (2–10)	10 (4–17.5)	7.0 (2–12)	0.19	0.27
Chronic migraine subtype, *n* (%)	16 (35.6)	14 (34.1)	2 (50)	49 (39.8)	0.61	0.61
Chronic migraine with MOH group	5 (11.1)	4 (9.8)	1 (25)	12 (9.8)	0.80	0.39
Median MMDs/MHDs (IQR)	12.0 (8–28)	12 (8–28)	28 (16–28)	16.0 (8–28)	0.73	0.11
Mean MMDs/MHDs	16.6 (9.0)	15.9 (8.9)	24 (8)	16.9 (9.2)	0.84	0.09
Median Thai-version MSQ v. 2.1 score (IQR)	58.6 (47.2–70.0)	61.5 (47.2–77.2)	51.5 (45–57.9)	62.9 (49.3–80.0)	0.48	0.32
Median Thai-version MIDAS score (IQR)	5 (5–10)	5 (0–10)	3 (0–11.3)	1 (0–6)	0.12	0.73
Number of preventive medications, *n* (%) ∙ Monotherapy ∙ Polytherapy	37 (82.2) 8 (17.8)	34 (82.9) 7 (17.1)	3 (75) 1 (25)	95 (77.2) 28 (22.8)	0.18	0.56
Migraine prevention inhibiting spreading depolarizations used, *n* (%)	18 (40)	18 (43.9)	0 (0)	47 (38.2)	0.97	NA
**Treatment outcomes**						
At 3 months (*n*)	45	41	4	123		
Median MMDs/MHDs (IQR)	5.0 (3.0–12.0)	5 (3–12)	7.5 (2–15.3)	6.0 (2.0–12.0)	0.73	0.86
Mean MMDs/MHDs (SD)	8.4 (7.4)	8.4 (7.5)	8.3 (6.9)	9.1 (8.7)	0.61	0.98
Mean reduction from baseline of MMDs/MHDs (SD)	8.3 (10.3)	7.6 (10.2)	15.8 (8.8)	7.9 (8.9)	0.79	0.13
30% reduction from baseline of MMDs/MHDs	32 (71.1)	28 (68.3)	4 (100)	81 (65.9)	0.65	NA
50% reduction from baseline of MMDs/MHDs	27 (60.0)	24 (58.5)	3 (75)	71 (57.7)	0.93	0.64
Median Thai-version MSQ v. 2.1 score (IQR)	72.1 (61.6–89.7)	74.4 (63.3–89.7)	61.5 (49–92.2)	81.5 (64.4–92.9)	0.16	0.51
Mean improvement from baseline of Thai-version MSQ v. 2.1 (SD)	15.4 (19.3)	15.3 (18.9)	16.1 (26)	14.3 (21.3)	0.80	0.94
Median Thai-version MIDAS score (IQR)	0 (0–4.0)	0 (0–4)	1.5 (1–7.3)	4.2 (0–3.0)	0.92	0.22
Mean reduction from baseline of Thai-version MIDAS score (SD)	3.5 (6.7)	3.8 (6.5)	1.5 (9.0)	1.0 (16.5)	0.41	0.54
**Treatment outcomes**						
At 6 months (*n*)	28	24	4	89		
Median MMDs/MHDs (IQR)	3.0 (2.0–10.0)	3 (2–10)	2.5 (2–21.8)	5.0 (2.5–10.0)	0.25	0.78
Mean MMDs/MHDs (SD)	7.1 (7.9)	6.9 (7.2)	8.8 (12.8)	7.3 (7.4)	0.91	0.67
Mean reduction from baseline of MMDs/MHDs (SD)	8.8 (9.6)	7.7 (8.9)	15.3 (12.5)	8.2 (8.2)	0.75	0.15
30% reduction from baseline of MMDs/MHDs	22 (78.6)	19 (79.2)	3 (75)	62 (69.7)	0.50	>0.99
50% reduction from baseline of MMDs/MHDs	18 (64.3)	15 (62.5)	3 (75)	52 (58.4)	0.74	0.55
Median Thai-version MSQ v. 2.1 score (IQR)	81.5 (54.3–94.4)	82.2 (53.3–93)	57.2 (54.3–57.5)	80.0 (65.8–91.9)	0.80	>0.99
Mean improvement from baseline of Thai-version MSQ v. 2.1 (SD)	16.3 (21.3)	15.7 (20.8)	20.5 (29.3)	12.8 (21.5)	0.49	0.73
Median Thai-version MIDAS score (IQR)	4.0 (0–13.3)	3 (0–14)	7 (0–7)	0 (0–4.0)	0.16	0.93
Mean reduction from baseline of Thai-version MIDAS score (SD)	1.0 (10.6)	0.9 (10.9)	1.3 (10.4)	1.6 (9.8)	0.81	0.96
**Treatment outcomes**						
At most recent follow-up median duration, years (IQR)	0.8 (0.3–2.8)	0.5 (0.25–2.5)	3 (1.9–4.5)	1.0 (0.5–3.0)	0.98	0.037*
Median MMDs/MHDs (IQR)	4.0 (2.0–9.8)	4 (2–9.8)	4 (1.3–16.5)	4.0 (1.0–8.3)	0.99	0.86
Mean MMDs/MHDs (SD)	6.2 (6.7)	6.1 (6.6)	7.3 (8.8)	6.9 (8.2)	0.60	0.75
Mean reduction from baseline of MMDs/MHDs (SD)	10.5 (9.1)	9.9 (9.0)	16.8 (9.2)	9.9 (8.8)	0.70	0.16
30% reduction from baseline of MMDs/MHDs	36 (81.8)	33 (82.5)	3 (75)	93 (76.2)	0.58	0.56
50% reduction from baseline of MMDs/MHDs	32 (72.7)	29 (72.5)	3 (75)	84 68.9)	0.77	>0.99
Median Thai-version MSQ v. 2.1 score (IQR)	82.9 (60.4–98.7)	83 (52.6–99)	80.1 (65.1–94)	85.8 (71.5–97.2)	>0.99	>0.99
Mean improvement from baseline of Thai-version MSQ v. 2.1 (SD)	19.3 (24.7)	18.3 (25.5)	28.2 (15.8)	18.4 (21.9)	0.83	0.45
Median Thai-version MIDAS score (IQR)	0 (0–2.5)	0 (0–3)	0 (0–0.75)	0 (0–2.0)	0.49	0.69
Mean reduction from baseline of Thai-version MIDAS score (SD)	4.8 (12.6)	4.9 (13.2)	4.5 (5.7)	3.1 (11.9)	0.46	0.96

MIDAS, migraine disability assessment questionnaire; MMDs/MHDs, monthly migraine/headache days; MOH, medication-overuse headache; MSQ, migraine-specific quality of life questionnaire.

**TABLE 3:** Correlation between complexity of aura and preventive treatment response.

At 3 months	Simple aura (*n* = 41)	Complex aura (*n* = 4)	Contingency coefficient	*P*
30% reduction from baseline of MMDs/MHDs	28	4	0.20	0.18
	13	0		
50% reduction from baseline of MMDs/MHDs	24	3	0.10	0.52
	17	1		
At 6 months	(*n* = 24)	(*n* = 4)		
30% reduction from baseline of MMDs/MHDs	19	3	0.04	0.85
	5	1		
50% reduction from baseline of MMDs/MHDs	15	3	0.10	0.63
	9	1		
At most recent follow-up	(*n* = 40)	(*n* = 4)		
30% reduction from baseline of MMDs/MHDs	33	3	0.06	0.71
	7	1		
50% reduction from baseline of MMDs/MHDs	29	3	0.02	0.92
	11	1		

MIDAS, migraine disability assessment questionnaire; MMDs/MHDs, monthly migraine/headache days; MOH, medication-overuse headache; MSQ, migraine-specific quality of life questionnaire.

**TABLE 4:** Binary logistic regression of treatment outcomes with an adjustment for migraine subtypes, aura subtypes, number of preventive medications used and comorbidity.

Variable	*P*	Exp (B)	95% CI for Exp (B)	
**30% reduction from baseline of MMDs/MHDs at 3 months**				
Complex aura	>0.99	2.00^9^	0.00	–
Comorbidity presence	0.07	5.16	0.90	29.48
Chronic migraine with/without MOH	0.82	1.48	0.05	43.86
Polytherapy for migraine prevention	0.21	0.25	0.29	2.17
History of migraine headache	0.63	1.02	0.94	1.12
MMDs/MHDs	0.97	0.99	0.82	1.21
**50% reduction from baseline of MMDs/MHDs at 3 months**				
Complex aura	0.61	1.95	0.15	24.93
Comorbidity presence	0.18	2.58	0.64	10.43
Chronic migraine with/without MOH	0.50	2.98	0.13	69.16
Polytherapy for migraine prevention	0.80	0.79	0.12	5.06
History of migraine headache	0.25	1.05	0.96	1.15
MMDs/MHDs	0.74	1.03	0.86	1.23
**30% reduction from baseline of MMDs/MHDs at 6 months**				
Complex aura	0.59	2.75	0.07	106.38
Comorbidity presence	0.12	11.52	0.54	247.24
Chronic migraine with/without MOH	0.85	1.60	0.01	195.65
Polytherapy for migraine prevention	0.46	0.30	0.01	7.22
History of migraine headache	0.21	1.09	0.96	1.23
MMDs/MHDs	0.38	1.16	0.84	1.59
**50% reduction from baseline of MMDs/MHDs at 6 months**				
Complex aura	0.35	4.75	0.18	127.39
Comorbidity presence	0.18	4.80	0.50	46.52
Chronic migraine with/without MOH	0.78	1.75	0.04	80.38
Polytherapy for migraine prevention	0.67	0.58	0.05	6.88
History of migraine headache	0.12	1.11	0.97	1.27
MMDs/MHDs	0.49	1.09	0.85	1.41
**30% reduction from baseline of MMDs/MHDs at most recent follow-up**				
Complex aura	0.93	1.14	0.08	16.57
Comorbidity presence	0.51	1.99	0.26	15.31
Chronic migraine with/without MOH	0.09	1918.86	0.32	116^7^
Polytherapy for migraine prevention	0.55	0.40	0.02	8.37
History of migraine headache	0.46	1.04	0.94	1.16
MMDs/MHDs	0.08	1.54	0.94	2.50
**50% reduction from baseline of MMDs/MHDs at most recent follow-up**				
Complex aura	0.62	1.90	0.15	23.62
Comorbidity presence	0.63	1.50	0.29	7.73
Chronic migraine with/without MOH	0.11	160.94	0.30	85897.04
Polytherapy for migraine prevention	0.96	1.07	0.07	16.61
History of migraine headache	0.50	1.03	0.94	1.14
MMDs/MHDs	0.09	1.34	0.96	1.89

MIDAS, migraine disability assessment questionnaire; MMDs/MHDs, monthly migraine/headache days; MOH, medication-overuse headache; MSQ, migraine-specific quality of life questionnaire.

## 4. Discussion

**FIGURE 2:**
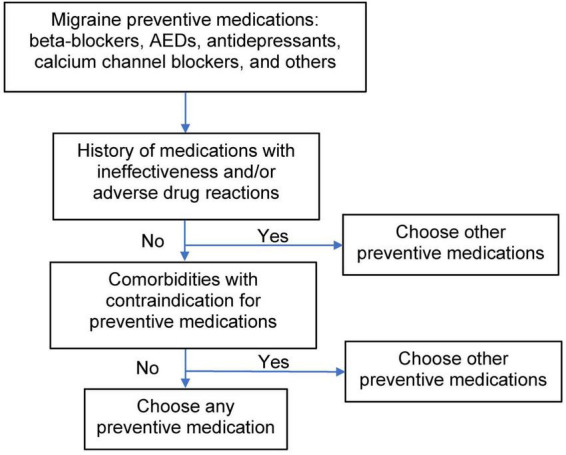
Decision tree for preventive treatment of migraine. AEDs, antiepileptic drugs.

In the present study, migraine aura was found in 54 patients (24.1%), which is consistent with the reported approximate range of from 15 to 33% of patients with migraine (Lucas, 2021). Comorbidities or coexisting diseases were found in 119 patients with migraine (53.1%) and needs to take into consideration when making the choice for migraine preventive oral treatments. Comorbidities may one reason for the low proportion of beta-blocker prescribing at the first visit to our headache center. Migraine preventive oral treatments have some limitations in their effectiveness and potential adverse drug reactions (Marín-Gracia et al., 2021). In practice, choice of a migraine preventive treatment involves a consideration of the history of the effectiveness and adverse drug reaction to previous or current migraine preventives, as well as comorbidities that contraindicate some preventive medications (Figure 2). In Thailand, 3 types of anti-CGRP monoclonal antibodies, which are used parenterally, are currently available for migraine prevention and may fill the gaps of ineffectiveness, adverse drug reactions, and contraindications of migraine preventives administered orally. However, the anti-CGRP monoclonal antibody treatments are costly. The proportions of migraine patients with aura and without aura who did not require migraine prevention because their migraine was infrequent was nearly the same and may imply a similar severity of disease in those with migraine with or without aura. Migraine patients with aura were younger than those without, which may imply that these patients perceive aura as a serious condition that warrants medical advice. Migraine patients with complex aura seemed to be more debilitated, and to include a higher proportion of those with medication overuse headache and comorbidity than those with simple aura and those without aura. However, the number of migraine patients with complex aura was small. Cortical spreading depression, which is proposed to cause migraine aura, may theoretically respond well to antiepileptic drugs. The present study found nearly half of migraine patients with simple aura, but not migraine with complex aura received antiepileptic drugs. One-quarter to four-fifths of migraine patients with simple or complex aura or without aura received monotherapy for migraine prevention. In clinical practice, monotherapy for migraine prevention should attempted first. If there is no response to monotherapy, polytherapy could be reasonably considered.

We found no significant differences in treatment outcomes between migraine patients with and without aura. The proportion of patients with a 30% reduction from baseline of MMDs/MHDs at 3 months follow-up tended to be higher in patients with aura (71.1%) than in patients without (65.9%) and the mean reduction from baseline of the Thai-version MIDAS score was 3.5 for those with migraine with aura and 1.0 for those without aura; however, the difference was not significant. At 6 months follow-up, there was a trend for improvement in the 30 and 50% reduction from baseline of MMDs/MHDs in patients with aura, of 78.6 and 64.3%, respectively, compared with patients without aura, namely 69.7 and 58.4%, respectively, and mean improvement from baseline of the Thai-version MSQ version 2.1 in patients with aura, with a mean score of 16.3 compared with 12.8 in patients without aura, and a similar trend was found for the most recent follow-up. Between aura subtypes, simple and complex aura, the mean reduction from baseline of MMDs/MHDs at 3, 6 months, and at most recent follow-up tended to be higher in patients with complex aura, at means of 15.8, 15.3, and 16.8 days, than in patients with simple aura, at means of 7.6, 7.7, and 9.9 days, respectively, and the proportion of patients with a 30 and 50% reduction from baseline of MMDs/MHDs at 3 months follow-up tended to be higher in patients with complex aura (100 and 75%) than in patients with simplex aura (68.3 and 58.5%); however, the difference was not significant. At 6 months follow-up, there was a trend to improvement in the 50% reduction from baseline of MMDs/MHDs in patients with complex aura, namely 75%, compared with patients with simple aura, at 62.5%, and mean improvement from baseline of the Thai-version MSQ version 2.1 score in patients with complex aura, with a mean score of 20.5 compared with 15.7 in patients with simple aura. We also adjusted for potential treatment effect modifiers, including chronic migraine subtype with/without medication-overuse headache (Geppetti et al., 2010), the number of preventive medications (Sacco et al., 2020), as well as comorbidity, aura subtypes, history of migraine headache, and MMDs/MHDs. We analyzed the correlation between the complexity of aura and preventive treatment response with a very weak, and weak relationship. One observational study conducted with data from 49 patients with migraine with aspirin 80 mg daily, found the aura frequency was reduced in 39 (93%) of 42 patients and complete cessation of aura in 20 (48%) (Turk et al., 2017). A retrospective study conducted with data from 203 patients with migraine with aura found 95 (46.8%) used acetylsalicylic acid and reported a significant reduction in aura duration (Anoaica et al., 2014). An open-label study including 59 patients with migraine with aura treated prospectively with lamotrigine for 3 years found that lamotrigine reduced both the frequency and duration of migraine aura (Lampl et al., 2005). Another open-label study including 16 patients with migraine with aura treated with levetiracetam found a reduction in migraine frequency and complete disappearance of aura in 7 (43%) (Brighina et al., 2006). Another open-label study with amiloride found reduced aura and headache symptoms in 4 of 7 patients (Holland et al., 2012). A fourth small-scale open-label study including 50 women with migraine with typical aura and migraine aura without headache showed a reduced number of attacks and aura duration (D’Andrea et al., 2009). A relatively large 6-month open-label plus 6-month double-blind, placebo-controlled randomized trial with *post hoc* analysis comparing migraine patients with (*n* = 269) and without aura (*n* = 542) to determine the efficacy of topiramate in preventing migraine aura found migraine without aura decreased by 43.1% in the final 28 days of the open-label phase, in which migraine aura decreased by 54.1% in migraine patients with aura, and a 44.3% reduction in migraines in those without aura. However, in the double-blind phase, while migraine was increased with placebo as it was in the full study, the increase versus the frequency after topiramate treatment was not significant, presumably due to a lack of statistical power in the subgroup analysis. Likewise, the investigators found no significant change in aura frequency between the groups. They concluded topiramate reduces the frequency of aura in parallel with migraine frequency, similarly for migraine patients with and without aura (Reuter et al., 2010).

Limitations of the present study may include its single-site design and sample size. If a larger multicenter study or if a meta-analysis is used to investigate the relationship between preventive treatment response and migraine aura subtypes, trends seen in the present study may become significant. Consistent use of a structured interview following the Migraine Aura Complexity Score and a more careful assessment of headaches as migrainous may provide a better classification of migraine aura.

Other limitations are as follows: we had recorded no data regarding the frequency of migraine aura and aura subtypes, and our inability to identify from patient records, migraine with comorbid tension-type headache or other primary headache disorders.

We found preventive treatment response tended to have more favorable outcomes in those with aura, especially those with complex aura.

## Data availability statement

The raw data supporting the conclusions of this article will be made available by the authors, without undue reservation.

## Ethics statement

The studies involving human participants were reviewed and approved by Institutional Review Board of the Faculty of Medicine, Chulalongkorn University. Written informed consent for participation was specifically waived for this retrospective study in accordance with the national legislation and the institutional requirements.

## Author contributions

TA contributed substantially to the acquisition and analysis of data. Both authors contributed substantially to the concept and design of the study, to interpretation of data, drafted the manuscript and revised it critically for important intellectual content, approved the version submitted, its publication, and agree to be accountable for all aspects of the work.
